# Memorials of the Craft of Surgery

**Published:** 1886-12

**Authors:** 


*Memorials of the Craft of Surgery.* By JOHN FLINT SOUTH. Edited by D'ARCY POWER, M.A., F.R.C.S. Pp. 412. London: CASSELL & CO. 1886.

This interesting work is the outcome of ten years' laborious investigations by John Flint South. His abundant notes from and copies of ancient manuscripts have been carefully and skilfully edited by Mr. D'Arcy Power, and his work is now before the public in this octavo volume. As Sir James Paget puts it in the Introduction, these Memorials "are not full records of the progress of Surgery in the present usual meaning of the words. They do not tell much of its progress as either a science or an art. . . . They are, properly, as the title says, 'Memorials of the *Craft* of Surgery'—that is, of its business and of the corporate life and government of those who, in successive centuries, practised it." In other words, the work might not inaptly be named "Memorials of the *Craftiness* of Surgeons." The diplomacy and finesse with which surgeons, originally disjointed free lances, gathered themselves into a compact phalanx, excluding obnoxious or unskilled rivals, securing charters, reproving misdemeanants, and upholding their own respectability, may be spoken of as craftiness just as aptly as craft. For instance, more than two centuries ago the Company of Barber-Surgeons showed the world how to escape the disagreeable necessity of paying their debts by becoming bankrupt. And many other curious facts go to create an impression, that in the erection of the magnificent fabric of the Royal College of Surgeons, crafty manœuvres were more potent than surgical craft. Human nature has changed but little since those days; much of it may even now be found among the chiefs of this great College.

Sir James Paget, in the Introduction, protests just a little against the vulgar error of supposing that English surgeons are, in any fair sense, the descendants of barbers. Of the ills that flesh is heir to, the small ones are by far the most grievous. The clumsiness and the deftness of good and of bad barbers have probably given as much pain and certainly more pleasure than surgery under similar circumstances has given. There is a science in hair, as there is in teeth. We have surgeon-dentists, with diplomas of the College of Surgeons; why should not that august body return to its old allegiance, resuscitate barber-surgeons, and enrich its coffers with their diploma fees? And the chiropodists too—they might claim admission, though we fear their friends the orthopædists might object. A man will be none the worse a surgeon for being able deftly to shave a chin, or extract a tooth, or pare a corn; and there can be no doubt that barbers, dentists, and chiropodists would be all the better artists for the possession of the diploma of the College of Surgeons. Ambrose Paré himself was a barber-surgeon: we need not be ashamed of our descent.

